# A Nomogram for Predicting Survival in Patients With Breast Cancer Liver Metastasis: A Population-Based Study

**DOI:** 10.3389/fonc.2021.600768

**Published:** 2021-06-02

**Authors:** Yu Xiong, Xia Shi, Qi Hu, Xingwei Wu, Enwu Long, Yuan Bian

**Affiliations:** Personalized Drug Therapy Key Laboratory of Sichuan Province, Department of Pharmacy, Sichuan Provincial People’s Hospital, School of Medicine, University of Electronic Science and Technology of China, Chengdu, China

**Keywords:** breast cancer liver metastasis, nomogram, AJCC-TNM stage, overall survival, prognosis

## Abstract

**Objective:**

The prognosis of patients with breast cancer liver metastasis (BCLM) was poor. We aimed at constructing a nomogram to predict overall survival (OS) for BCLM patients using the SEER (Surveillance Epidemiology and End Results) database, thus choosing an optimized therapeutic regimen to treat.

**Methods:**

We identified 1173 patients with BCLM from the SEER database and randomly divided them into training (n=824) and testing (n=349) cohorts. The Cox proportional hazards model was applied to identify independent prognostic factors for BCLM, based on which a nomogram was constructed to predict 1-, 2-, and 3-year OS. Its discrimination and calibration were evaluated by the Concordance index (C-index) and calibration plots, while the accuracy and benefits were assessed by comparing it to AJCC-TNM staging system using the decision curve analysis (DCA). Kaplan-Meier survival analyses were applied to test the clinical utility of the risk stratification system.

**Results:**

Grade, marital status, surgery, radiation therapy, chemotherapy, CS tumor size, tumor subtypes, bone metastatic, brain metastatic, and lung metastatic were identified to be independent prognostic factors of OS. In comparison with the AJCC-TNM staging system, an improved C-index was obtained (training group: 0.701 *vs.* 0.557, validation group: 0.634 *vs.* 0.557). The calibration curves were consistent between nomogram-predicted survival probability and actual survival probability. Additionally, the DCA curves yielded larger net benefits than the AJCC-TNM staging system. Finally, the risk stratification system can significantly distinguish the ones with different survival risk based on the different molecular subtypes.

**Conclusion:**

We have successfully built an effective nomogram and risk stratification system to predict OS in BCLM patients, which can assist clinicians in choosing the appropriate treatment strategies for individual BCLM patients.

## Introduction

Breast cancer is the most common cancer in women around the world and the second leading cause of cancer death after lung cancer in American women (1). Breast cancer can metastasize to bone, lung, liver, pleura, skin, soft tissue, etc. (2). Among them, breast cancer liver metastases (BCLM) are very common in the clinical treatment of breast cancer. Approximately 50% of all breast cancer will occur with metastasis and the liver represents the third most frequent site of metastasis in patients with breast cancer (3, 4). Additionally, BCLM is considered the most lethal compared with other sites of metastases (e.g., the lung, bone, or brain), with 5-year survival rates of only 3.8-12% (median survival, 4-21 months) (5). Despite systemic chemotherapy including hormonal therapy, biological therapy, palliative therapy, and radiation having been performed, the prognoses of BCLM remains poor with a median survival of only 4.8-15 months (6, 7). Besides, some patients may exhibit resistance to endocrine therapy, and some may demonstrate a poor response to chemotherapy, and the latter accounts for much of the high mortality in patients with BCLM (3, 8). However, a special forecasting tool for BCLM is lacking. Nomograms are considered to be reliable and convenient prognostic tools, and are widely used for prognostication in oncology because of their quantitative analysis of risk variables (9, 10). Thus, in this study, we propose to construct nomograms for predicting overall survival (OS) in patients with BCLM.

In the study, we used the latest data available in the SEER (Surveillance Epidemiology and End Results) population-based database. We have three objectives. First, we described the demographics and clinicopathologic characteristics of the population. Second, significant variables related to BCLM were picked out to establish the prognostic model. Third, we constructed nomograms for visualizing the model and predicting the survival of BCLM. With the help of this aiding tool, more optimized therapeutic regimen might be chosen clinically, thus helping patients obtain a better prognosis.

## Materials and Methods

### Study Design and Patients Selection

**Figure 1:**
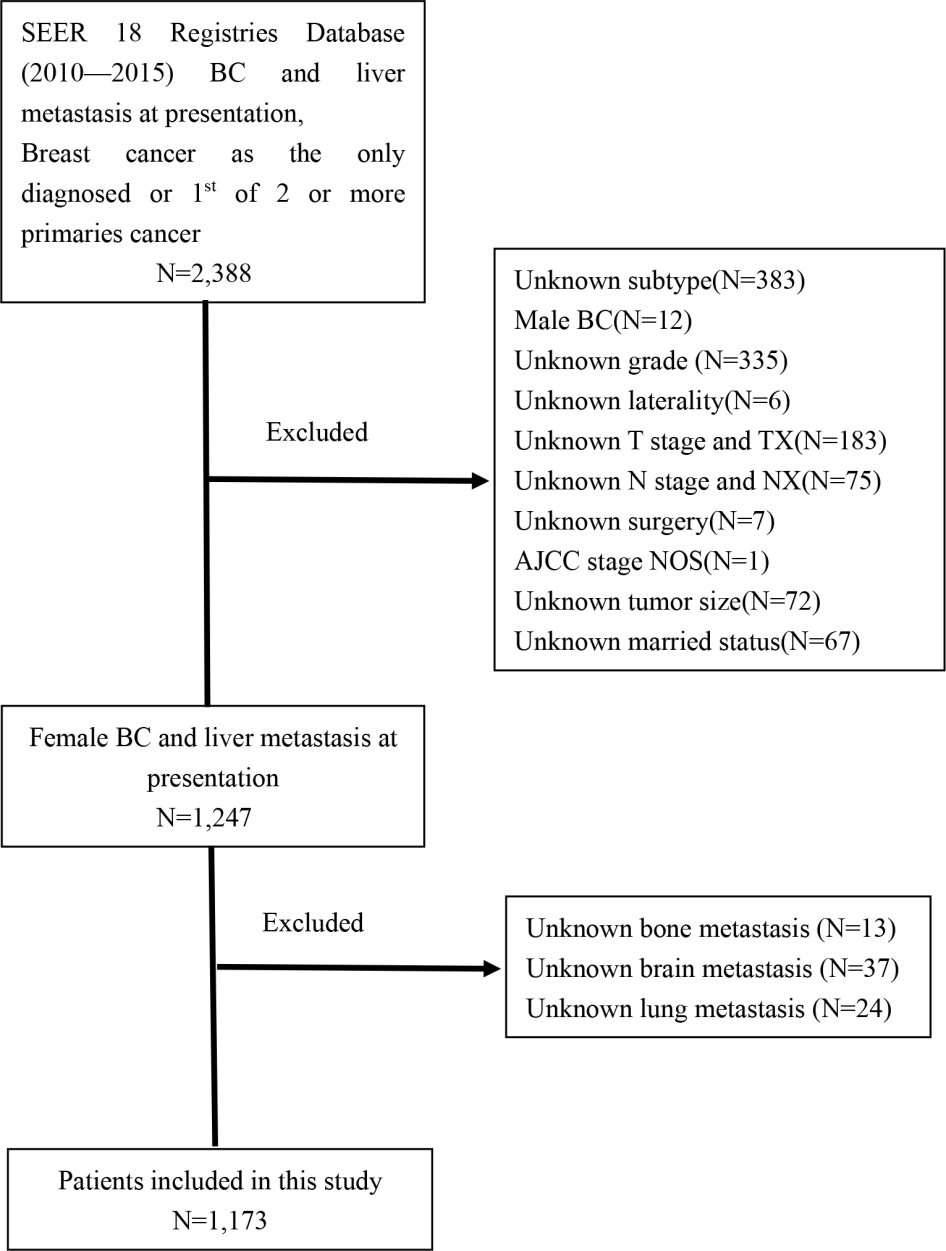
The flowchart of patients identified in the study.

The patients included in this study were retrieved from the SEER 18 database by using SEER*Stat program version 8.3.5, which is a public national registry database containing data on cancer occurrences in 18 areas of United States and representing approximately 34.6% of the population. The trial population encompassed adult female breast cancer patients with liver metastases diagnosed from 2010 to 2015 because information about the molecular subtypes and sites of distant metastasis was collected in 2010. The inclusion criteria included patients who had a known history of breast cancer, active follow-up, and breast cancer as the only diagnosed or 1^st^ of 2 or more primary cancers. We excluded patients with unknown subtype, male BC, and those who did not have complete information (grade unknown, laterality unknown, AJCC stage unknown, TNM stage unknown, surgery unknown, tumor size unknown, married status unknown, and metastatic sites unknown). A flow chart of the selection is shown in **Figure 1**. Eventually, we identified 1,173 eligible patients for this study. No formal consent was required for this type of retrospective study.

### Statistical Analysis

All these patients were randomly divided into 7:3 training and validation groups. Univariate COX Proportional Hazard Regression analysis was developed to identify independent prognostic factors to construct prognostic factors. Based on the results of the univariate analysis (P value<0.1), multivariate COX Proportional Hazard Regression analysis was performed to build nomograms with significant variables (P value<0.05) in the training group. We employed 1-,2-, and 3-years OS for analysis in the nomogram. Concordance index (C-index) and the calibration curves were used to evaluate the discriminative and accuracy ability of the nomogram. Both discrimination and calibration were evaluated by bootstrapping 1000 times. Otherwise, decision curve analysis (DCA) was employed to evaluate the benefits and advantages of our new predicting model over other existing tools (for example, 8th edition AJCC TNM staging system) (11). Furthermore, a risk stratification model was developed on the aggregate score of every patient in the nomogram, which was distributed into two prognostic groups (low and high) according to its median value.

All of these statistical methods were performed using R software version 3.6.3 (http://www.r-project.org) and Empower (R) (www.empowerstats.com, XY Solutions, inc.Boston MA). Statistical significance was set at P<0.05 in a two-tailed test.

## Results

### Patient Characteristics

A total of 1,173 female patients with BCLM were evaluated from 2010 to 2015 (1,173 patients for a primary cohort:824 patients for a training cohort and 349 for a validation cohort). The median follow-up time of the entire cohort was 18 months, and 1-, 2-, 3- year survival rates were 0.66, 0.41, 0.23, respectively. In the training cohort, more than half of the patients were over 56 years old (51.3%), white (72.9%), diagnosed between 2013 and 2015 (51.3%) and unmarried (50.4%). Moreover, Luminal A, which was the most common subtype of BCLM, was poorly differentiated (representing Grade III and IV) in 61.6 and 59.6% in the training and testing cohorts, respectively. Furthermore, the proportion of chemotherapy-received patients was much larger than the surgery and radiation therapy, 71.8%, 31.4%, and 28.4% in the training cohort, respectively. Additionally, in patients with BCLM, the incidence of bone metastatic was the highest (56.9%), and the lung metastatic was the second (34.6). The detailed demographics and clinicopathologic characteristics of the 3 cohorts were presented in **Table 1**.

**Table 1:** Demographics and clinicopathologic characteristics of the cohort with BCLM.

Variables	Total cohort	Training cohort	Validation cohort
	No. (%)	No. (%)	No. (%)
Year of diagnosis			
2010-2012	590 (50.3)	401 (48.7)	189 (54.2)
2013-2015	583 (49.7)	423 (51.3)	160 (45.8)
Age			
18—56 years	570 (48.6)	401 (48.7)	169 (48.4)
≥56 years	603 (51.4)	423 (51.3)	180 (51.6)
Race			
White	840 (71.6)	601 (72.9)	239 (68.5)
Black	203 (17.3)	127 (15.4)	76 (21.8)
Other	130 (11.1)	96 (11.7)	34 (9.7)
Marital status			
Married	566 (48.3)	409 (49.6)	157 (45.0)
Unmarried	607 (51.7)	415 (50.4)	192 (55.0)
Grade			
Well differentiated	457 (39.0)	316 (38.4)	141 (40.4)
Poorly differentiated	716 (61.0)	508 (61.6)	208 (59.6)
Laterality			
Left	630 (53.7)	453 (55.0)	177 (50.7)
Right	543 (46.3)	371 (45.0)	172 (49.3)
T stage			
T1	118 (10.1)	94 (11.4)	24 (6.9)
T2	408 (34.8)	275 (33.4)	133 (38.1)
T3	235 (20.0)	164 (19.9)	71 (20.3)
T4	412 (35.1)	291 (35.3)	121 (34.7)
N stage			
N0	213 (18.2)	155 (18.8)	58 (16.6)
N1	626 (53.4)	435 (52.8)	191 (54.7)
N2	157 (13.4)	105 (12.7)	52 (14.9)
N3	177 (15.1)	129 (15.7)	48 (13.8)
Surgery			
No	807 (68.8)	565 (68.6)	242 (69.3)
Yes	366 (31.2)	259 (31.4)	107 (30.7)
Radiation therapy			
No	833 (71.0)	590 (71.6)	243 (69.6)
Yes	340 (29.0)	234 (28.4)	106 (30.4)
Chemotherapy			
No	324 (27.6)	232 (28.2)	92 (26.4)
Yes	849 (72.4)	592 (71.8)	257 (73.6)
CS tumor size			
<50mm	610 (52.0)	426 (51.7)	184 (52.7)
≥50mm	563 (48.0)	398 (48.3)	165 (47.3)
CS Tumor Size/Ext Eval			
0	370 (31.5)	268 (32.5)	102 (29.2)
1—6	803 (68.5)	556 (67.5)	247 (70.8)
Tumor subtypes			
Luminal A	501 (42.7)	338 (41.0)	163 (46.7)
Luminal B	271 (23.1)	199 (24.2)	72 (20.6)
HER2 enriched	200 (17.1)	142 (17.2)	58 (16.6)
Triple-negative breast cancer	201 (17.1)	145 (17.6)	56 (16.1)
Bone metastatic			
No	505 (43.1)	362 (43.9)	142 (41.0)
Yes	668 (56.9)	462 (56.1)	206 (59.0)
Brain metastatic			
No	1089 (92.8)	766 (93.0)	323 (92.5)
Yes	84 (7.2)	58 (7.0)	26 (7.5)
Lung metastatic			
No	767 (65.4)	535 (64.9)	232 (66.5)
Yes	406 (34.6)	289 (35.1)	117 (33.5)

HER2, Human epidermal growth factor receptor 2. For marital status, unmarried consists of single, divorced, separated, and widowed. For race, ‘other’ includes American Indian, AK Native, Asian, and Pacific Islander. Laterality is defined as the laterality of tumor primary sites. For grade, well differentiated including Grade Ⅰ and Ⅱ, poorly differentiated including Grade Ⅲ and Ⅳ.

### Univariate and Multivariate COX Hazard Regression Analysis

The hazard ratios (HR) for OS according to all variables in the univariate and multivariate COX proportional hazard model are shown in **Table 2**. The univariate COX-Regression analysis demonstrated that age at diagnosis, race, marital status, grade, N stage, surgery, radiation therapy, chemotherapy, CS tumor size, CS tumor size/Ext Eval, tumor subtypes, bone metastatic, brain metastatic, and lung metastatic were associated with OS. All of these factors were entered the multivariate COX-Regression analysis, in which marital status, grade, surgery, radiation therapy, chemotherapy, CS tumor size, tumor subtypes, bone metastatic, brain metastatic, and lung metastatic were found to be final prognostic factors. These variables were further used to construct the nomogram.

**Table 2:** Univariate and multivariate COX regression analysis based on all variables for OS.

Variables	Univariate Analysis		Multivariate Analysis		Points
	HR (95% CI)	P Value	HR (95% CI)	P Value	
Year of diagnosis					
2010-2012	Reference		—	—	—
2013-2015	0.94 (0.78,1.12)	0.4750	—	—	—
Age					
18—56 years	Reference		Reference		—
≥56 years	1.49 (1.26, 1.77)	<0.0001	1.20 (1.00, 1.45)	0.0545	—
Race					
White	Reference		Reference		—
Black	1.31 (1.05, 1.64)	0.0161	1.14 (0.90, 1.44)	0.2903	—
Other	0.98 (0.74, 1.30)	0.8865	1.16 (0.87, 1.56)	0.3142	—
Marital status					
Married	Reference		Reference		0
Unmarried	1.42 (1.20, 1.69)	<0.0001	1.25 (1.05, 1.49)	0.0144	23
Grade					
Well differentiated	Reference		Reference		0
Poorly differentiated	1.21 (1.02, 1.45)	0.0322	1.34 (1.09, 1.65)	0.0047	19
Laterality					
Left	Reference		—	—	—
Right	0.95 (0.80, 1.13)	0.5526	—	—	—
T stage					
T1	Reference		Reference		—
T2	0.99 (0.74, 1.32)	0.9190	1.02 (0.76, 1.38)	0.8758	—
T3	1.02 (0.75, 1.40)	0.8779	0.78 (0.51, 1.18)	0.2417	—
T4	1.29 (0.97, 1.71)	0.0760	0.92 (0.64, 1.32)	0.6570	—
N stage					
N0	Reference		Reference		—
N1	0.70 (0.56, 0.87)	0.0013	0.80 (0.63, 1.01)	0.0610	—
N2	0.70 (0.52, 0.95)	0.0206	0.81 (0.59, 1.13)	0.2171	—
N3	0.80 (0.61, 1.06)	0.1215	0.78 (0.57, 1.05)	0.1055	—
Surgery					
No	Reference		Reference		35
Yes	0.60 (0.49, 0.72)	<0.0001	0.62 (0.49, 0.78)	<0.0001	0
Radiation therapy					
No	Reference		Reference		31
Yes	0.83 (0.69, 1.00)	0.0558	0.74 (0.60, 0.91)	0.0050	0
Chemotherapy					
No	Reference		Reference		100
Yes	0.46 (0.38, 0.55)	<0.0001	0.48 (0.39, 0.59)	<0.0001	0
CS tumor size					
<50mm	Reference		Reference		0
≥50mm	1.32 (1.11, 1.56)	0.0015	1.37 (1.05, 1.80)	0.0220	15
CS Tumor Size/Ext Eval					
0	Reference		Reference		—
1—6	0.79 (0.66, 0.95)	0.0112	1.09 (0.89, 1.33)	0.4118	—
Tumor subtypes					
Luminal A	Reference		Reference		0
Luminal B	0.65 (0.51, 0.81)	0.0002	0.75 (0.59, 0.96)	0.0246	32
HER2 enriched	0.69 (0.53, 0.89)	0.0046	0.91 (0.68, 1.21)	0.5223	63
Triple-negative breast cancer	2.27 (1.82, 2.83)	<0.0001	2.89 (2.24, 3.74)	<0.0001	95
Bone metastatic					
No	Reference		Reference		0
Yes	1.45 (1.22, 1.72)	<0.0001	1.47 (1.22, 1.78)	<0.0001	34
Brain metastatic					
No	Reference		Reference		0
Yes	2.19 (1.62, 2.95)	<0.0001	1.58 (1.14, 2.19)	0.0064	48
Lung metastatic					
No	Reference		Reference		0
Yes	1.75 (1.47, 2.08)	<0.0001	1.38 (1.15, 1.65)	0.0006	38

HER2, Human epidermal growth factor receptor 2. For marital status, unmarried consists of single, divorced, separated, and widowed. For race, ‘other’ includes American Indian, AK Native, Asian and Pacific Islander. Laterality is defined as the laterality of tumor primary sites. For grade, well differentiated including Grade Ⅰ and Ⅱ, poorly differentiated including Grade Ⅲ and Ⅳ.

### Calibration and Validation of the Nomogram

**Figure 2:**
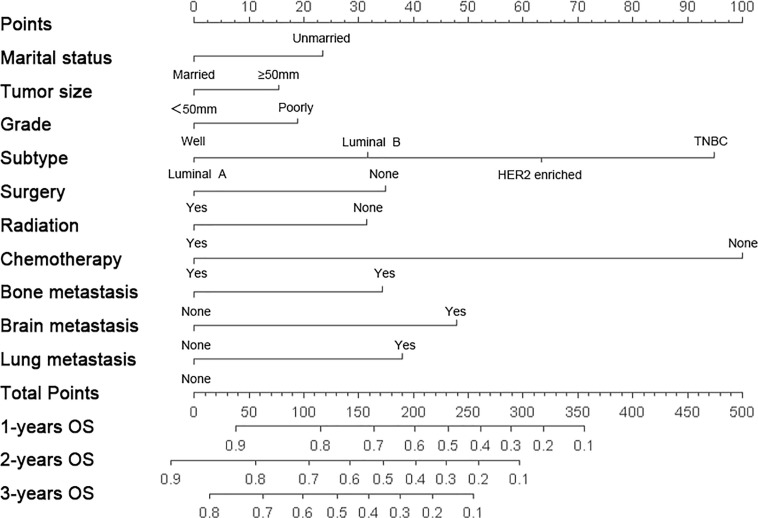
Nomograms for predicting 1-, 2-, 3-year overall survival (OS) for female patients with breast cancer liver metastasis (BCLM). HER2, Human epidermal growth factor receptor 2; TNBC, triple-negative breast cancer.

**Figure 3:**
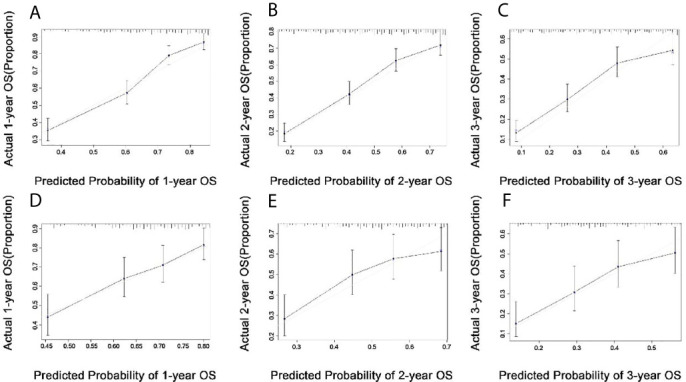
Calibration plots in the training **(A–C)** and validation **(D–F)** cohorts for 1-year, 2-year, and 3-year overall survival (OS).

**Figure 4:**
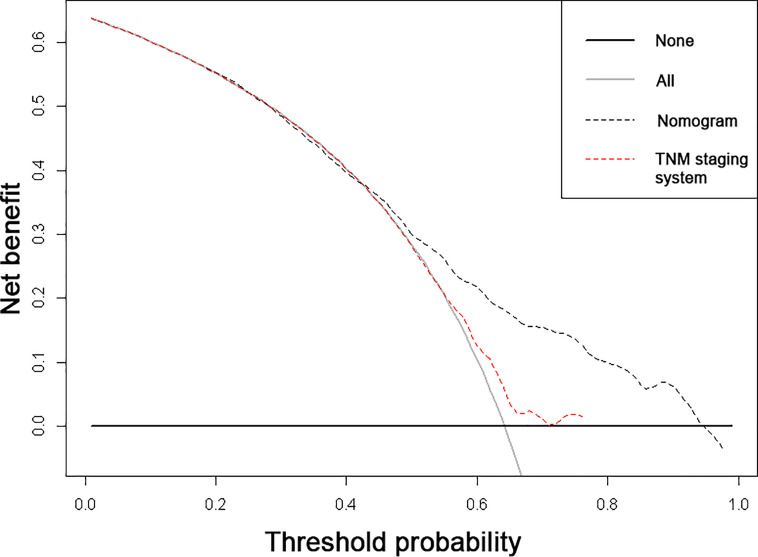
Decision curve analyses (DCA) of the nomogram and 7^th^ edition AJCC-TNM staging system for 3-year overall survival. The x-axis represents the threshold probabilities, and the y-axis measures the net benefits. The horizontal line parallel to the axis shows that overall death occurred in no patients, while the solid grey line demonstrates that all patients will have overall death at a specific threshold probability. The black and red dashed line represents the nomogram and 7^th^ edition AJCC-TNM staging system, respectively.

The nomogram was constructed to predict 1-year, 2-year, and 3-year overall survival of patients with these ten significantly independent factors (**Figure 2**). The score of each category was given on the point scale axis (**Table 2**). The nomogram showed that chemotherapy contributed the most to prognosis, followed by tumor subtype and brain metastasis. A total score could be easily obtained by adding each single score of the selected variables, and then projecting the total score to the bottom scale can estimate the probabilities of 1-, 2-, and 3-year OS for each individual patient to some extent. The C-index of nomogram (training group=0.701, validation group=0.634) was higher than that of seventh version AJCC-TNM staging system (0.557), which demonstrated that the model had an acceptable predictive accuracy. The calibration plots of the nomogram showed excellent agreement in the training cohort and satisfactory agreement in the validation cohort between the actual observations and the predicted outcomes (**Figure 3**). Besides, decision curve analysis (DCA) was performed to compare the clinical application and benefits of the nomogram with that of the AJCC-TNM staging system. This analysis was performed to evaluate 3-year OS of BCLM patients. As shown in **Figure 4**, DCA analyses significantly demonstrated the growth of net benefits of the new model over 7^th^ version AJCC-TNM staging system with wide and practical ranges of threshold probabilities.

### Risk Stratification System

**Figure 5:**
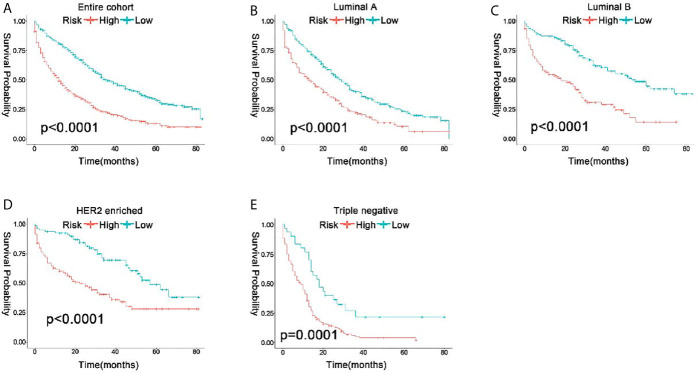
Kaplan-Meier curve to test the stratification system between the entire cohort **(A)** and each subtype **(B–E)**.

Because these results showed excellent prediction efficiency in survival of the nomogram, we calculated total points based on the predicted score calculated by the nomogram. According to the cutoff value (median points), all the patients were separated into low risk (total points <171.95) and high risk (total points ≥171.95) groups. In the entire cohort, 2-year OS rate of patients with low risk, and high risk were 0.55 and 0.28. The 581 low-risk patients had significantly better OS than the 592 high-risk patients (P<0.0001) by Kaplan-Meier analyses (**Figure 5A**). Furthermore, as molecular subtype was an important prognostics factor for OS, we stratified the patients on the basis of their ER, PR, and HER 2 statuses to figure out the effects of the risk stratification system. From the study cohort, the patients in Luminal A, Luminal B, HER2 enriched, and Triple-negative breast cancer were 501, 271, 200, and 201 cases, respectively. Regardless of the patients’ subtype, high-risk groups had much worse outcomes than low-risk groups (P<0.05) (**Figures 5B–E**). Ultimately, all these results proved the robust prognostic value of the risk stratification system among molecular subtype.

## Discussion

As is well known, BCLM is a heterogeneous disease characterized by diverse histopathologic and molecular features, which are associated with distinct clinical outcomes (12). There are remarkable advances in system treatment, the prognosis of patients with BCLM is dismal (13). For example, traditional palliation locoregional treatment [transarterial embolization (TAE), and transarterial chemoembolization (TACE)] often combined with personalized drug therapy to treat BCLM (4, 14). Therefore, establishing a model to predict the risk for BCLM is necessary, which can aid the development of therapeutic strategies for these patients. Although the 7th AJCC-TNM staging system is acceptable for predicting the prognosis in BCLM patients, it neglects some important variables such as marital status, age, and race, etc. (14, 15). Thus, in this study, we constructed a more comprehensive model for better prediction of prognosis in BCLM patients. In order to better understand the use of this nomogram, we can take a patient with BCLM as an example. A married woman with 62mm liver metastases from breast cancer, grade IV, luminal B, received radiation and chemotherapy without surgery, and no metastases beyond the liver. The patient has approximately 82%, 67% and 55% survival probability the first, second and third year, respectively. This well-development clinical nomogram is a good decision-tool, which can be used to predict the outcome of an individual, bringing benefits to both clinicians and patients.

### Prognostic Factors of Patients With BCLM

By COX regression analyses, we identified marital status, grade, surgery, radiation therapy, chemotherapy, CS tumor size, tumor subtypes, bone metastatic, brain metastatic, and lung metastatic as independent predictors of overall survival. A previous study by Lin et al. has shown that sex, age at diagnosis, grade, N stage, ER status, PR status, and HER2 status can be risk factors for BCLM (8). Yang et al. have reported that HER2 status, tumor size, and lymph node metastasis were independent prognostic factors for survival in BCLM (16). It is obvious that tumor subtype is a significant risk factor for OS of patients with BCLM because we can choose molecular targeted therapy or endocrine therapy for the corresponding molecular subtype, which can greatly improve the prognosis (17). Our study found that married patients have better prognosis than the unmarried ones, which is not shown in other studies. The reason for this may be that single patients are faced with more distress, depression, and anxiety than married counterparts. Moreover, the adherence with prescribed treatment is associated with marital status. Married patients are more likely to follow treatment than unmarried ones, which may have a better control of BCLM (18). It was shown that tumor differentiation was an independent factor for predicting overall survival in similar reports, which was consistent with our results (19). However, contrary to other studies, age is not an important prognostic factor in our study. The small amount of data is one possible reason. Other factors mentioned above, radiation therapy, chemotherapy, and metastases other than liver metastases were also identified as significant predictors of prognosis. These results were consistent with many previous reports (16, 18–20).

### Predictive Efficacy of the Nomograms

We constructed nomograms based on the Cox proportional hazards model for visualizing survival. The nomograms were validated internally and their performance was evaluated by calibration and discrimination. In the present study, the calibration curves performed optimal agreement in predicting OS, which guaranteed the reliability of the established nomograms. Also, the C-index was much higher compared with the 7^th^ AJCC-TNM staging system (0.701 *vs* 0.557), suggesting the high discrimination ability of the nomogram. According to the previous studies regarding the BCLM, the C-index was between 0.6 and 0.8, indicating that our nomograms showed a moderate predictive effect on prognosis (5, 8, 19). In addition, DCA also showed that our nomograms have potentially higher predictive value regarding prognosis. The nomograms showed that chemotherapy contributed the most to prognosis, the patients without chemotherapy had a much worse prognosis than those who had chemotherapy treatment. Also, the patients in TNBC suffered from the worst prognosis among all the molecular subtypes, which is consistent with other studies (17, 20, 21).

Our outcomes also indicated the magnitude of poor prognosis as the tumor grade changed from well to poorly differentiated. Moreover, the idea of constructing a risk stratification system to verify the robust prognosis of nomograms is novel. All in all, our nomograms can make an accurate estimate for prognosis of patients with BCLM. And this was a rare study that constructed a visual prediction model aiming at improving the survival rate of patients with BCLM and it provided such useful information.

### Limitations

Nevertheless, this study has several limitations. First, because of the lack of information on the treatment of liver metastasis, some common treatment options, such as transarterial embolization (TAE), transarterial chemoembolization (TACE), and selective internal radiotherapy (SIRT) were not included in this study (22, 23). Second, SEER database did not record variables such as occupation, education, and family history, which may potentially affect the results derived from the Cox proportional hazard model (24). Third, our study was definitely a retrospective analysis, so the hypotheses raised remained to be proven in future investigation with larger data volume. At last, the drug information is also one of the important factors that we need to consider, as some studies presented that low doses of paclitaxel enhanced liver metastasis of breast cancer cells in the mouse model (25 **).**

## Conclusion

The current study comprehensively analyzed the prognosis of patients with BCLM on the basis of the SEER population level database, and constructed a nomogram for accessing the individualized survival estimates for patients with BCLM. The outcome showed that marital status, grade, surgery, radiation therapy, chemotherapy, CS tumor size, tumor subtypes, bone metastatic, brain metastatic, and lung metastatic are considered to be the ten independent risk factors. We have confirmed the excellent and clinical application of the nomograms by comparing them to the 7^th^ AJCC-TNM staging system.

## Data Availability Statement

Publicly available datasets were analyzed in this study. This data can be found here: https://seer.cancer.gov/.

## Author Contributions

YX designed this research. YX, XS, and QH conducted analyses of the statistics and drafted the manuscript. QH and XW carried out collection of data and processed the figures or tables. All of the authors reviewed the manuscript. All authors contributed to the article and approved the submitted version.

## Funding

This work was supported by The National Key Specialty Construction Project of Clinical Pharmacy (NO.30305030698), and Clinical Research and Transformation Project of Sichuan provincial People’s Hospital (No. 2018LY09).

## Conflict of Interest

The authors declare that the research was conducted in the absence of any commercial or financial relationships that could be construed as a potential conflict of interest.

## References

[1] DeSantis CE Ma J Gaudet MM Newman LA Miller KD Goding Sauer A. Breast Cancer Statistics, 2019. CA Cancer J Clin (2019) 69:438–51. 10.3322/caac.21583 31577379

[2] Mathew A Rajagopal PS Villgran V Sandhu GS Jankowitz RC Jacob M. Distinct Pattern of Metastases in Patients With Invasive Lobular Carcinoma of the Breast. Geburtshilfe Frauenheilkd (2017) 77:660. 10.1055/s-0043-109374 28757653 PMC5489406

[3] Treska V Cerna M Liska V Treskova I Narsanska A Bruha J. Surgery for Breast Cancer Liver Metastases–Factors Determining Results. Anticancer Res (2014) 34:1281–6. 24596373

[4] Bale R Putzer D Schullian PJC. Local Treatment of Breast Cancer Liver Metastasis. Cancers (2019) 11:1341. 10.3390/cancers11091341 PMC6770644 31514362

[5] Ruiz A Wicherts DA Sebagh M Giacchetti S Castro-Benitez C Van Hillegersberg R. Predictive Profile-Nomogram for Liver Resection for Breast Cancer Metastases: An Aggressive Approach With Promising Results. Ann Surgical Oncol (2017) 24:535–45. 10.1245/s10434-016-5522-7 27573523

[6] Ma R Feng Y Lin S Chen J Lin H Liang X. Mechanisms Involved in Breast Cancer Liver Metastasis. J Transl Med (2015) 13:1–10. 10.1186/s12967-015-0425-0 25885919 PMC4440291

[7] Goddard ET Fischer J Schedin P. A Portal Vein Injection Model to Study Liver Metastasis of Breast Cancer. J Vis Exp (2016) 118:e54903. 10.3791/54903 PMC5226462 28060292

[8] Lin Z Yan S Zhang J Pan Q. A Nomogram for Distinction and Potential Prediction of Liver Metastasis in Breast Cancer Patients. J Cancer (2018) 9:2098–106. 10.7150/jca.24445 PMC6010683 29937928

[9] Balachandran VP Gonen M Smith JJ Dematteo RP. Nomograms in Oncology: More Than Meets the Eye. Lancet Oncol (2015) 16:e173–80. 10.1016/S1470-2045(14)71116-7 PMC4465353 25846097

[10] Xiong Z Deng G Huang X Li X Xie X Wang J. Score for the Survival Probability in Metastasis Breast Cancer: A Nomogram-Based Risk Assessment Model. Cancer Res Treat (2018) 50:1260–9. 10.4143/crt.2017.443 PMC6192925 29334609

[11] Vickers AJ Elkin EB. Decision Curve Analysis: A Novel Method for Evaluating Prediction Models. Med Decis Making (2006) 26:565–74. 10.1177/0272989X06295361 PMC2577036 17099194

[12] Brodt P. Liver Metastasis: Biology and Clinical Management. Berlin: Springer Science & Business Media (2011).

[13] Ji L Cheng L Zhu X Gao Y Fan L Wang Z. Risk and Risk and Prognostic Factors of Breast Cancer With Liver Metastases. BMC Cancer (2021) 21:238. 10.1186/s12885-021-07968-5 33676449 PMC7937288

[14] Chen S Liu Y Yang J Liu Q You H Dong Y. Development and Validation of a Nomogram for Predicting Survival in Male Patients With Breast Cancer. Front Oncol (2019) 9:361. 10.3389/fonc.2019.00361 31139562 PMC6527749

[15] Plichta JK Ren Y Thomas SM Greenup RA Fayanju OM Rosenberger LH. Implications for Breast Cancer Restaging Based on the 8th Edition AJCC Staging Manual. Ann Surg (2020) 271:169–76. 10.1097/SLA.0000000000003071 PMC6588495 30312199

[16] Yang A Xiao W Zheng S Kong Y Zou Y Li M. Predictive Nomogram of Subsequent Liver Metastasis After Mastectomy or Breast-Conserving Surgery in Patients With Nonmetastatic Breast Cancer. Cancer Control (2020) 28:1073274821997418. 10.21203/rs.3.rs-16000/v1 PMC8482719 33626925

[17] Xie J Xu Z. A Population-Based Study on Liver Metastases in Women With Newly Diagnosed Breast Cancer. Cancer Epidemiol Biomarkers Prev (2019) 28:283–92. 10.1158/1055-9965.EPI-18-0591 30487134

[18] Wu Q Wang W-J Huang Y-Q Fang S-Y Guan Y-J. Nomograms for Estimating Survival in Patients With Liver-Only Colorectal Metastases: A Retrospective Study. Int J Surg (2018) 60:1–8. 10.1016/j.ijsu.2018.10.032 30366096

[19] Chen QF Huang T Shen L Wu P Huang ZL Li W. Prognostic Factors and Survival According to Tumor Subtype in Newly Diagnosed Breast Cancer With Liver Metastases: A Competing Risk Analysis. Mol Clin Oncol (2019) 11:259–69. 10.3892/mco.2019.1890 PMC6667840 31396386

[20] Insa A Lluch A Prosper F Marugan I Martinez-Agullo A Garcia-Conde J. Prognostic Factors Predicting Survival From First Recurrence in Patients With Metastatic Breast Cancer: Analysis of 439 Patients. Breast Cancer Res Treat (1999) 56:67–78. 10.1023/A:1006285726561 10517344

[21] Wyld L Gutteridge E Pinder S James J Chan S Cheung K. Prognostic Factors for Patients With Hepatic Metastases From Breast Cancer. British J Cancer (2003) 89:284–90. 10.1038/sj.bjc.6601038 PMC2394248 12865918

[22] Hortobagyi GN. Trastuzumab in the Treatment of Breast Cancer. New Eng J Med (2005) 353:1734–5. 10.1056/NEJMe058196 16236745

[23] Damian S Tessari A Capri G Mariani P Bianchi GV Mariani G. Hepatic Trans-Arterial Chemoembolization (TACE) in Metastatic Breast Cancer. Am Soc Clin Oncol (2013) 31(15_suppl):e12017–e12017. 10.1200/jco.2013.31.15_suppl.e12017

[24] Doll KM Rademaker A Sosa JA. Practical Guide to Surgical Data Sets: Surveillance, Epidemiology, and End Results (SEER) Database. JAMA Surg (2018) 153:588–9. 10.1001/jamasurg.2018.0501 29617544

[25] Li Q Ma Z Liu Y Kan X Wang C Su B. Low Doses of Paclitaxel Enhance Liver Metastasis of Breast Cancer Cells in the Mouse Model. FEBS J (2016) 283:2836–52. 10.1111/febs.13767 27307301

